# The omega-3 DHA induces pyroptosis and mitochondrial dysfunction in ovarian cancer cells via ROS and caspase-1 activation

**DOI:** 10.1038/s41420-025-02854-6

**Published:** 2026-01-14

**Authors:** Gabriel Pasquarelli-do-Nascimento, Sarah Pinho Bezerra, Júlia Perin Manchine, Nathalia Cristina Silva Lago, Heloísa Antoniella Braz-de-Melo, Nathalia Soares da Cruz, Paula Maria Quaglio Bellozi, Amanda Pereira Rocha, Igor de Oliveira Santos, Fernanda Gomes Lago, Sabrina Azevedo Machado, André Moraes Nicola, Andreza Fabro de Bem, Sônia Nair Báo, Kelly Grace Magalhães

**Affiliations:** 1 Laboratory of Immunology and Inflammation, Department of Cell Biology, University of Brasilia, Brasilia, DF Brazil; 2 Department of Physiological Sciences, University of Brasília (UnB), Brasilia, Brazil; 3 Faculty of Medicine, University of Brasília (UnB), Brasilia, Brazil; 4 Laboratory of Molecular Immunology, Rockefeller University, New York, NY USA; 5 INCT - National Institute of Science and Technology, INCT - Human Pathogenic Fungi, Sao Paulo, Brazil; 6 Laboratory of Microscopy and Microanalysis, Department of Cell Biology, University of Brasilia, Brasilia, DF Brazil; 7 INCT - National Institute of Science and Technology, INCT - Mucosa and Skin, Brasilia, Brazil

**Keywords:** Cell biology, Cancer

## Abstract

Ovarian cancer remains one of the most lethal gynecologic malignancies due to late diagnosis, limited treatment options, and frequent chemoresistance. Docosahexaenoic acid (DHA), a long-chain omega-3 polyunsaturated fatty acid, has been associated with anti-tumor effects in various cancer models. Here, we investigated the effects of DHA on cell death, oxidative stress, and mitochondrial function in A2780 human ovarian cancer cells. Our data show that DHA decreases cell viability and proliferation in a dose- and time-dependent manner, promoting lytic cell death with increased membrane permeability and LDH release. We identified pyroptosis as the predominant death mechanism, evidenced by caspase-1 activation, pore formation, and mitochondrial dysfunction. DHA treatment rapidly increased intracellular reactive oxygen species (ROS) and mitochondrial superoxide levels, which were essential for both membrane pore formation and the loss of mitochondrial membrane potential. Notably, ROS scavenging with N-acetylcysteine reversed DHA-induced mitochondrial damage and pyroptosis, indicating ROS dependence. Furthermore, DHA reduced mitochondrial content and impaired spare respiratory capacity, suggesting disrupted mitochondrial adaptability. Caspase-1 inhibition restored both mitochondrial integrity and respiratory function, highlighting a mechanistic role for caspase-1 in mediating DHA-induced bioenergetic dysfunction. Collectively, our findings reveal that DHA compromises ovarian cancer cell survival by triggering ROS- and caspase-1-dependent pyroptosis and mitochondrial dysfunction. This study expands the understanding of DHA’s anti-cancer mechanisms and positions it as a promising candidate for adjuvant therapies targeting mitochondrial vulnerabilities and immunogenic cell death pathways in ovarian cancer.

## Introduction

Ovarian cancer, the seventh most common cancer globally, has several risk factors, including genetics, infertility, nulliparity, endometriosis, obesity, and age [1, 2]. The primary therapeutic approaches for ovarian malignancies currently include cytoreductive surgery, radiotherapy, and chemotherapy with a platinum-taxane combination [3]. Furthermore, ovarian cancer is the second most frequent cause of death among gynecologic malignancies worldwide and is associated with a poor prognosis, with a 5-year survival rate of only 47% [1, 4]. The high mortality rate of ovarian cancer is largely due to its asymptomatic nature in the early stages, often leading to late-stage diagnosis, when survivorship is significantly lower [5–7]. Additionally, elevated recurrence rates following surgery and chemotherapy contribute to the high number of deaths associated with this disease [8, 9]. Understanding the global incidence of ovarian cancer is essential for recognizing the substantial challenge it presents to public health systems. These facts highlight the urgency of investigating means of attenuating the devastating impact of ovarian cancer.

Along with eicosapentaenoic acid (EPA) and α-linoleic acid (ALA), docosahexaenoic acid (DHA) is a polyunsaturated fatty acid (PUFA) of the omega-3 family that has long been associated with several benefits, including central nervous system health maintenance, cardiovascular protection, metabolic homeostasis, immune system adequate function, blood coagulation control, and neoplasm development modulation [10–17]. Studies investigating the impact of DHA in the context of cancer show that this lipid can modulate intracellular pathways linked with cell cycle progression, invasiveness, and death [15–30]. A previous study by our group demonstrated that DHA can induce pyroptosis in human breast cancer cells [31], a highly inflammatory form of programmed cell death that is gaining attention in cancer research. This process involves caspase-1 activation and early membrane disruption, which is marked by GSDMD cleavage and Lactate Dehydrogenase (LDH) release [32]. In cancer cells, this mechanism intersects with the roles of reactive oxygen species (ROS), which in augmented levels can influence mitochondrial function [33, 34].

Interestingly, docosahexaenoic acid (DHA) has emerged as a modulator of pyroptosis in cancer cells, promoting caspase-1 activation and enhancing the impact of ROS [35]. Although DHA has been implicated in augmented intracellular ROS levels and caspase-1 activation and pyroptosis occurrence in ovarian tumor models, the intricate relationship between oxidative status, mitochondrial function, and pyroptotic cell death in ovarian cancer cells treated with DHA remains unclear. Considering the DHA effects on cancer models, the present study aimed to investigate the impacts of DHA on death profile, oxidative status, and mitochondrial function of ovarian cancer cells in vitro.

## Results

### DHA reduces the viability and proliferation of ovarian cancer cells without major impact on the cell cycle

**Fig. 1:**
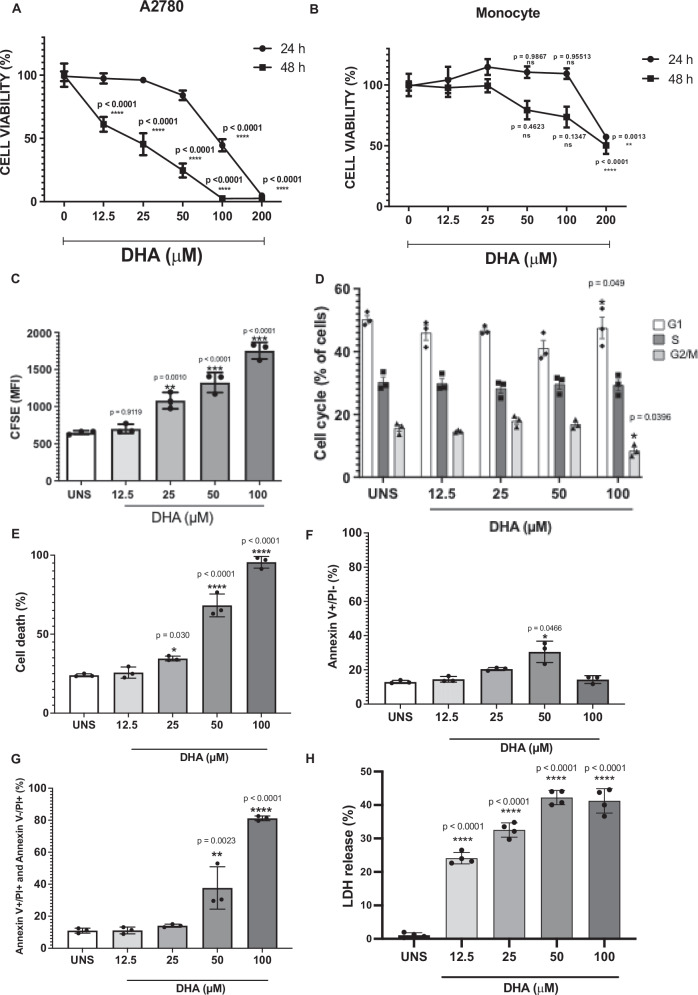
DHA reduces the viability and proliferation of ovarian cancer cells without major impact on the cell cycle. **A** MTT assay data suggest that DHA treatment of the human ovarian cancer cell A2780 for 24 (lines with black circle) and 48 h (lines with black square) copes with decreased cell viability in a time- and dose-dependent manner. **B** DHA treatment of non-tumor cells used as a control (PBMC-derived human monocytes), also for 24 and 48 h, leads to diminished cell viability only after 48 h of treatment at 200 µM. MTT assays’ findings are shown as a percentage of cell viability in comparison to unstimulated cells (UNS) (absence of DHA). **C** CFSE staining of A2780 cells for flow cytometry (FL-1 log scale) indicates that all tested DHA concentrations diminish cell proliferation after 24 h of stimulus. **D** Flow cytometry of A2780 cells stained with PI (FL-2 linear scale) suggests that only 24 h of treatment with 200 µM impacts cell cycle progression. Annexin-V and Propidium iodide (PI) double staining of A2780 cells for flow cytometry (FL-1 and FL-3 log scale) suggests that DHA treatment triggers A2780. **E** cell death, specifically **F** apoptosis and **G** lytic cell death after 24 h of stimulus. **H** DHA treatment leads to Lactate dehydrogenase (LDH) release at late time points. Cell viability, proliferation, death profile, cell cycle, and LDH levels data presented are the mean of 3 experiments ± standard deviation (SD). Statistical discrepancies in comparison to unstimulated cells (UNS) were represented with the asterisk (*) as it follows: *p* < 0.05 (one symbol), *p* < 0.01 (two symbols), *p* < 0.001 (three symbols), and *p* < 0.0001 (four symbols). Figures represent three independent replicates.

To assess the impact of DHA on the viability of human ovarian cancer cells (A2780), we measured mitochondrial activity using the MTT assay following 24- and 48-h treatments with increasing DHA concentrations. As shown in Fig. 1A, a 24-h treatment with 50, 100, and 200 µM DHA significantly reduced A2780 cell viability in a dose-dependent manner. This effect was even more pronounced after 48 h, when a lower concentration (12.5 µM) was sufficient to induce cytotoxicity. These results indicate that DHA decreases A2780 cell viability in both a time- and dose-dependent fashion. To determine whether this cytotoxic effect was specific to cancer cells, we treated PBMC-derived monocytes with DHA and observed that only the highest concentration tested (200 µM) induced cytotoxicity in these non-neoplastic cells (Fig. 1B). Since this concentration was not used in subsequent experiments, it suggests a degree of selectivity in DHA’s effects on A2780 cells.

Given DHA’s impact on cell viability, we next examined whether it also influenced A2780 cell proliferation. As shown in Fig. 1C and Supplementary Figs. 1A, B, A2780 cells exhibit significantly reduced proliferation after being treated with all DHA tested concentrations (12.5, 25, 50, and 100 µM) for 24, 48 and 72 h. However, only the highest dose (100 µM) led to cell cycle arrest after 24 h of treatment (Fig. 1D).

Since DHA treatment reduced A2780 cell viability and proliferation but only induced cell cycle arrest at 100 µM, we next investigated its effects on cell death. To analyze the cell death profile, we performed Annexin-V/Propidium Iodide (PI) double staining and assessed A2780 cells via flow cytometry. As shown in Fig. 1E, a 24-h treatment with 50 and 100 µM DHA significantly increased cell death. At 50 µM, DHA primarily induced apoptotic cell death (Fig. 1F). However, both 50 and 100 µM DHA treatments resulted in a notable increase in double-stained (Annexin-V + PI+) events, indicating a predominance of lytic cell death compared to untreated cells (Fig. 1G). This finding was further supported by increased LDH release following 24-h DHA exposure (Fig. 1H), reinforcing the occurrence of membrane rupture.

### DHA induces pyroptotic cell death in human ovarian cancer cells

**Fig. 2:**
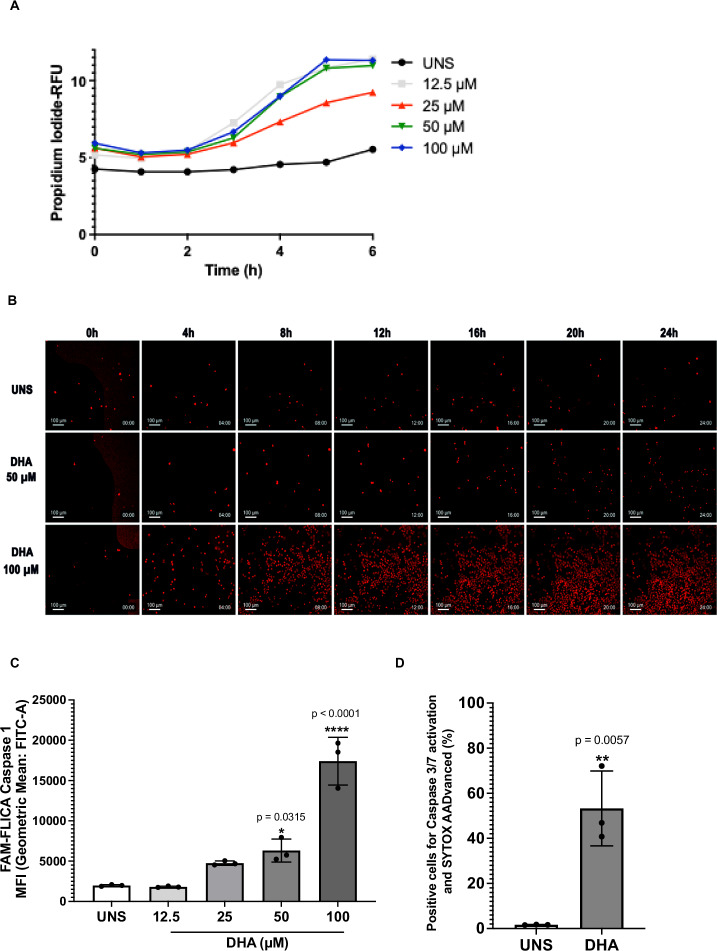
DHA induces pyroptotic cell death in human ovarian cancer cells. **A** PI uptake analysis indicates the occurrence of membrane integrity loss initiating at early time points. **B** Microscopic analysis of cells treated with DHA and exposed to PI indicates that pore formation is maintained in late time points, as 24 h. A2780 cell exposure to DHA leads to the activation of **C** caspase-1 and **D** caspase-3. The data presented are the mean of 3 experiments ± standard deviation (SD). Statistical discrepancies in comparison to unstimulated cells (UNS) were represented with the asterisk (*) as it follows: *p* < 0.05 (one symbol), *p* < 0.01 (two symbols), *p* < 0.001 (three symbols), and *p* < 0.0001 (four symbols). Figures represent three independent replicates.

Given that DHA induced lytic cell death, we assessed whether it triggered early membrane pore formation. Using the PI fluorescent probe, we observed significant membrane integrity disruption within the first 6 h of DHA treatment, as indicated by increased fluorescence in all DHA-treated conditions compared to the unstimulated group (Fig. 2A) and we visualized the occurrence of PI uptake due to membrane pore formation in early and late treatment time points (Fig. 2B and Supplementary Videos S1–S3), which suggests that DHA exerts cytotoxic effects on A2780 cells at both early and late time points.

Pyroptotic membrane pore formation is typically driven by caspase-1 activation. To determine whether DHA-induced cell death involves caspase-1, we quantified its activity using the FAM-FLICA caspase-1 probe. Our results revealed that DHA treatment displayed significant effects on caspase-1 activation after 24 h (Fig. 2C), supporting its role in pyroptosis. Additionally, as caspase-3 activation has been implicated in pyroptotic cell death in cancer models, we examined caspase-3 activity in A2780 cells using the FAM-FLICA caspase-3/7 probe and SYTOX AADvanced staining to identify lytic cell death. As shown in Fig. 2D, treatment with 50 µM DHA significantly increased caspase-3 activation in A2780 cells undergoing lytic cell death compared to unstimulated controls.

### DHA triggers ROS and mitochondrial superoxide production, leading to mitochondrial membrane potential loss

**Fig. 3:**
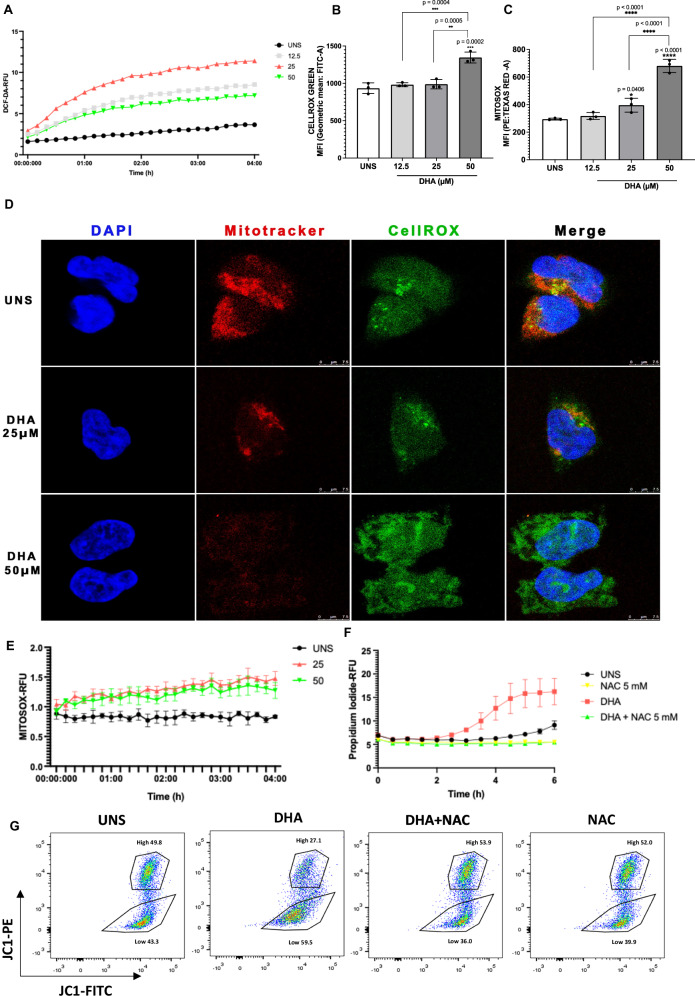
DHA triggers ROS and mitochondrial superoxide production, leading to mitochondrial membrane potential loss. **A** DCFDA A2780 staining for fluorescence analysis in a spectrophotometer indicates that DHA treatment copes with ROS production at initial time points. **B** CellROX Green staining of A2780 for flow cytometry (FL-1 log scale) suggests that DHA stimulation also leads to ROS generation after 24 h of treatment. **C** MitoSOX^TM^ Red staining for flow cytometry (FL-2 log scale) shows that DHA triggers mitochondrial superoxide production after 24 h of treatment. **D** Staining of A2780 cells with MitoTracker Red CMXRos and CellROX Green for confocal microscopic analysis suggests that DHA stimulation leads to augmented ROS generation and decreased number of mitochondria in A2780. **E** MitoSOX^TM^ Red staining for fluorescence analysis in a spectrophotometer indicates that DHA triggers mitochondrial superoxide production at early time points. **F** PI uptake assay suggests that A2780 cell pyroptotic death is dependent on increased ROS levels. **G** JC-1 staining for flow cytometry indicates that the effects of DHA on A2780 cell mitochondrial membrane potential depend on augmented ROS levels. The data presented are the mean of 3 experiments ± standard deviation (SD). Statistical discrepancies in comparison to unstimulated cells (UNS) were represented with the asterisk (*) and differences compared to other experimental groups were indicated by the number sign (#) as it follows *p* < 0.05 (one symbol), *p* < 0.01 (two symbols), *p* < 0.001 (three symbols), and *p* < 0.0001 (four symbols). Figures represent three independent replicates.

Since 50 µM was the lowest DHA concentration that induced caspase-1 activation, we used this dose as the highest tested concentration in subsequent analyses. Given the role of ROS in multiple cell death pathways, including pyroptosis, we assessed whether DHA treatment affected ROS production in A2780 cells. Using the fluorescent probe DCFDA, we observed that DHA rapidly induced ROS generation at early time points (Fig. 3A). This increase persisted after 24 h of treatment, as shown in Fig. 3B. We also verified that 24-h DHA treatment of A2780 led to elevated mitochondrial superoxide levels compared to unstimulated controls, as illustrated by Fig. 3C. The augmented levels of intracellular ROS and mitochondrial superoxide in cells treated with DHA for 24 h were associated with the reduction of the mitochondrial content of cells in a dose-dependent manner (Fig. 3D). We also detected that cells stimulated with 25 and 50 μM of DHA displayed an early increase in mitochondrial superoxide levels (Fig. 3E), indicating that DHA influenced intracellular and mitochondrial oxidative statuses at both early and late time points.

Considering our findings regarding the effects of DHA on the oxidative status of A2780 ovarian cancer cells, we sought to investigate whether increased ROS levels are directly linked to pyroptosis in A2780 ovarian cancer cells. As depicted by Fig. 3F, 5 mM NAC treatment of cells stimulated with 50 μM of DHA abolished the effects of DHA on early pore formation. Taking into consideration that DHA treatment coped with intracellular and mitochondrial ROS production, decreased mitochondrial content, and that the inhibition of ROS generation led to decreased pore formation in cells treated with DHA, we analysed whether the treatment with NAC influenced the impacts of DHA on mitochondrial membrane potential. We verified that cells treated with only 50 μM of DHA presented decreased percentage of cells with high mitochondrial potential (27.1%, 26% and 19.9%) compared to unstimulated cells (UNS) (49.8%, 48.6% and 47.1%) (Fig. 3G). In contrast, when ovarian cancer cells were stimulated with both DHA and NAC (53.9%, 49.2% and 53.7%), the effects of DHA on mitochondrial membrane potential were abolished, presenting augmented amounts of cells with high mitochondrial potential, comparable to unstimulated (UNS) cells.

### DHA reduces mitochondrial content and impairs spare respiratory capacity

**Fig. 4:**
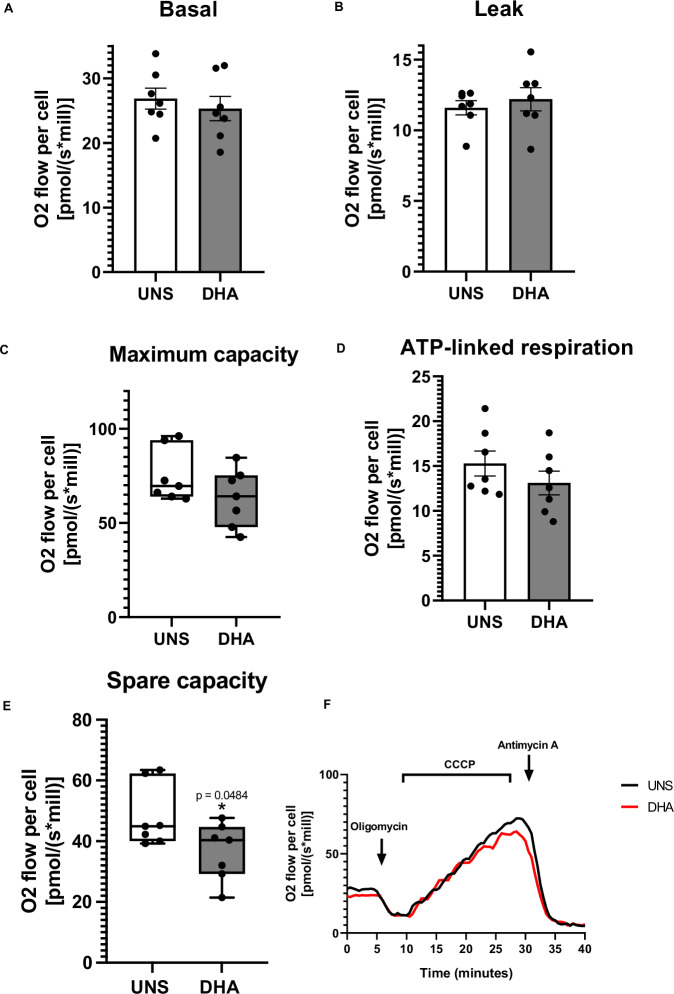
DHA reduces mitochondrial content and impairs spare respiratory capacity. High-resolution respirometry analyses of A2780 cells suggest that 50 µM of DHA does not impact on **A** basal respiration, **B** proton leak, **C** maximum respiratory capacity, **D** ATP-linked respiration, but impairs the **E** mitochondrial spare respiratory capacity after 24 h of treatment. ATP-synthase inhibitor oligomycin (0.1 µg/mL), CCCP (14.4–21.6 µM), rotenone (0.5 µM) and antimycin A (1 µM) were used for determining mitochondrial respiratory parameters. ATP-linked oxygen consumption was calculated by the mathematical difference between basal and proton-leaking OCR, and spare respiratory spare capacity by the difference between maximum capacity and basal OCR. The high-resolution respirometry data presented are the mean of 6 experiments ± standard deviation (SD). Statistical discrepancies in comparison to unstimulated cells (UNS) were represented with the asterisk (*) as it follows: *p* < 0.05 (one symbol). Figures represent three independent replicates. **F** High-resolution respirometry analysis representative figure.

Since ROS primarily originates from mitochondria, we assessed whether DHA treatment affected mitochondrial respiration in A2780 cells. After 24 h of stimulation with 50 µM DHA, we observed no significant changes in basal respiration (Fig. 4A), proton leak (Fig. 4B), maximal respiratory capacity (Fig. 4C), or ATP-linked respiration (Fig. 4D). However, DHA treatment significantly impaired the spare mitochondrial respiratory capacity (Fig. 4E) compared to unstimulated cells, suggesting a reduced ability of A2780 cells to meet increased energy demands. Figure 4F illustrates the oxygen consumption across all experimental replicates.

### Caspase-1 inhibition reverses DHA-induced pyroptosis and mitochondrial dysfunction

**Fig. 5:**
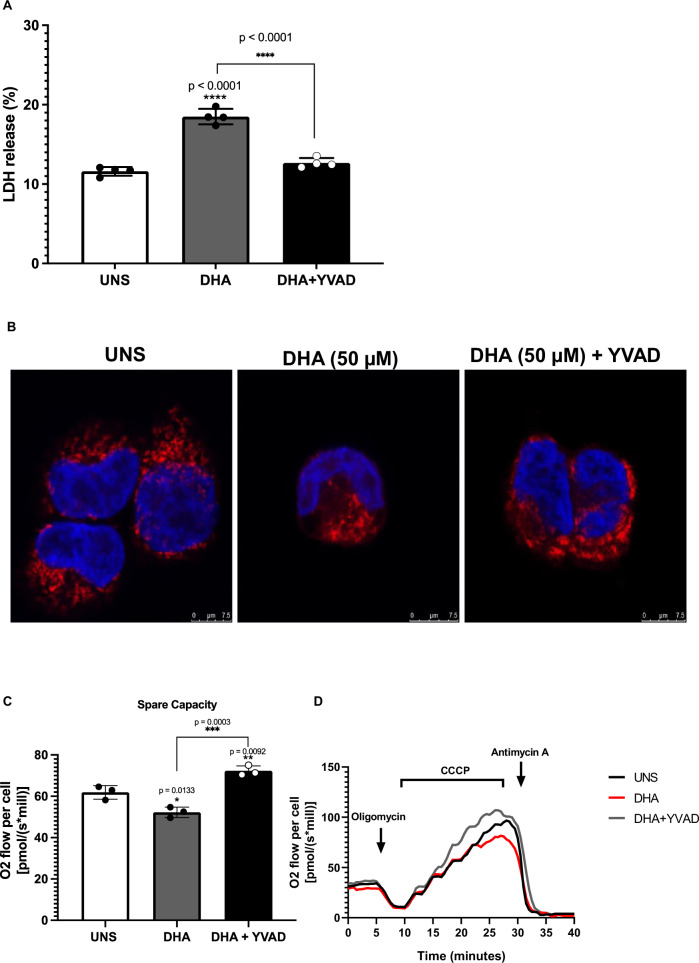
Caspase-1 inhibition reverses DHA-induced pyroptosis and mitochondrial dysfunction. **A** LDH release analysis of A2780 cells via spectrophotometry suggests that the treatment for 1 h with the caspase-1 specific inhibitor YVAD (20 µM) abrogates the effects of DHA on LDH release by these cells. We represented data as a percentage compared to the positive control (A2780 cells treated with the kit lysis buffer for 45 min before supernatant collection). The data presented are the mean of 4 experiments ± standard deviation (SD). **B** MitoTracker Red CMXRos staining of A2780 cells suggests that the inhibition of caspase-1 using YVAD abolishes the effects of DHA on cell mitochondrial content. DAPI/PBS solution was used for visualizing the cell nucleus compartment. Samples were analysed using confocal microscopy (*Leica TCS SP5*). 63× objective lenses were used for collecting the images. The data represent 3 independent experiments. **C** High-resolution respirometry analyses of A2780 cells suggest that the impairing impact of DHA on mitochondrial spare capacity is abolished by YVAD pre-treatment. ATP-synthase inhibitor oligomycin (0.1 µg/mL), CCCP (14.4 to 21.6 µM), rotenone (0.5 µM), and antimycin A (1 µM) were used for determining mitochondrial respiratory parameters. ATP-linked oxygen consumption was calculated by the mathematical difference between basal and proton-leaking OCR, and respiratory spare capacity by the difference between maximum capacity and basal OCR. The data presented are the mean of 3 experiments ± standard deviation (SD). Statistical differences in comparison to unstimulated cells (UNS) were represented with the asterisk (*) as follows: *p* > 0.05 (one symbol), *p* > 0.01 (two symbols), and *p* > 0.001 (three symbols). Statistical differences between other experimental groups were represented with a number sign (#) as it follows: *p* < 0.05 (one symbol), *p* < 0.01 (two symbols), and *p* < 0.001 (three symbols), and *p* < 0.0001 (four symbols). Figures represent three independent replicates. **D** High-resolution respirometry analysis representative figure.

To determine the role of caspase-1 in DHA-induced pyroptotic cell death in A2780 human ovarian cancer cells, we pretreated these cells with the specific caspase-1 inhibitor YVAD. As shown in Fig. 5A, while 24-h treatment with 50 µM DHA significantly increased LDH release, indicating lytic cell death, pre-treatment with YVAD markedly reduced extracellular LDH levels. These findings suggest that DHA-induced pyroptotic death in A2780 cells is caspase-1 dependent.

Given that caspase-1 inhibition prevented DHA-induced pyroptosis, we next investigated whether it also influenced mitochondrial content. DHA treatment at 50 µM reduced mitochondrial number in A2780 cells; however, as illustrated in Fig. 5B, caspase-1 inhibition abolished this effect. These results indicate that the impact of DHA on mitochondrial content is dependent on caspase-1 activation.

Since DHA treatment impaired A2780 mitochondrial spare respiratory capacity (SRC), and caspase-1 inhibition reversed its effects on mitochondrial number, we next examined whether caspase-1 inhibition could also restore mitochondrial respiration. As shown in Fig. 5C, D, A2780 cells pretreated with the caspase-1 inhibitor displayed a significant recovery in SRC compared to those treated with DHA alone. These findings suggest that caspase-1 activation is a key mediator of DHA-induced mitochondrial dysfunction, as its inhibition rescues both mitochondrial content and respiratory function in A2780 cells.

## Discussion

Although ovarian cancer is the seventh most frequent malignancy in the global population, it is a highly lethal disease [1]. A factor that exacerbates the impacts of ovarian tumors in global death statistics is the lack of efficient screening options for detecting early-stage ovarian tumors, as approximately 70% of women are diagnosed with grade III/IV disease, stages that correlate with high morbidity and mortality [36–39]. Natural compounds have been widely explored in the context of diverse cancer models. In addition to its positive effects on the individual immune system, inflammatory and metabolic statuses, cardiovascular function, blood coagulation, and liver and heart functioning [11–17], DHA has been extensively investigated in several tumor models. Authors link DHA with modulations in cell viability and death profile, oxidative status, and mitochondrial function [19–28, 30, 31, 35, 40–59].

Studies suggest that DHA triggers several phenomena that lead to cytotoxic effects in cancer cells that have not been reported to occur in non-transformed cells. Wang and collaborators described that DHA treatment decreases A2780 viability and invasiveness. They also described that DHA stimulation modulates the NF-κB pathway in these cells [41]. Authors investigating the specific cell death pathways triggered by DHA on cancer models have linked this lipid to autophagy and ferroptosis in vitro [21, 30, 40]. DHA has also been connected to apoptotic cell death occurrence in in vitro models of squamous cell carcinoma [21], neuroblastoma [23], leukemia [24, 42], multiple myeloma [27], and malignancies of the liver [22, 43], colon [20, 44, 45], lung [21, 25, 46], pancreas [26], stomach [47], breast [21, 28, 48], and ovary [19, 49]. In a previous study of our group, we reported that DHA treatment of the human breast cancer cells MDA-MB-231 coped with pyroptotic death in these cells in vitro [31]. Calbay and others discovered that DHA also triggers pyroptosis in various ovarian cancer cells in vitro, in a caspase-1 dependent manner [35]. Although various authors described that DHA triggers caspase-3 activation in cancer models, this was only reported to occur during apoptotic cell death [19–28, 42–49]. Herein, we presented for the first time that caspase-3 activation occurs during pyroptosis of A2780 cells treated with DHA. We encourage further analyses to dissect the influence of DHA-induced caspase-3 activation on A2780 pyroptosis.

In the present study, we described that the DHA-induced pyroptosis of A2780 cells occurred concomitantly with the generation of intracellular ROS and mitochondrial superoxide in these cancer cells, initiating at early time points. Several other studies connect DHA to cancer cell oxidative stress, as shown for in vitro models of glioma [50], squamous cell carcinoma [51], and tumors from colon [20, 52, 53], lung [25, 46], stomach [54], breast [28, 55, 56], prostate [57, 58], and ovary [51, 59]. Authors suggest that the elevated rate of ROS synthesis characteristic of transformed cells may explain why DHA triggers augmented ROS levels in these cells: DHA can easily undergo nonenzymatic lipid peroxidation and, thus, maintain ROS levels higher [10]. DHA was also described to influence the antioxidant capacity of cancer cells, as described by Merendino and collaborators, that showed that DHA leads to active glutathione (GSH) extrusion in a pancreatic cancer cell line [60], and by Ding and Lind, that discovered that DHA treatment of many ovarian cancer cell lines coped with diminished glutathione peroxidase-4 (GPx-4) protein expression [61]. Studies evaluating the effects of antioxidants will be key for determining the role of DHA on ROS production in A2780 cells.

Mainly produced in the mitochondria as side-products of the respiration process [62], excessive ROS have been linked by many studies to damage in intracellular lipids, RNA, DNA, and proteins [63], and to mitochondrial dysfunction [64]. Mitochondrial dysfunction can occur due to inadequate organelle number and dysregulated electron transport chain (ETC) function [65]. Tamarindo and others discovered that DHA leads to a loss of mitochondrial membrane potential in prostate cancer cells [58]. However, authors did not investigate other parameters that indicate mitochondrial function, such as the SRC. Herein, we showed for the first time that DHA treatment leads to decreased mitochondrial membrane potential and mitochondrial SRC in ovarian cancer cells. Defined as the mathematical difference between the basal and the maximum respiration, SRC reflects the mitochondrial property to produces energy during acute stress [66, 67]. SRC has been pictured as a robust and reproducible parameter for evaluating mitochondrial plasticity and adaptability [67, 68]. Thus, SRC can indicate the fitness of the mitochondrial intracellular network [69] and can indicate the occurrence of mitochondrial dysfunction invisible under basal conditions [70]. Therefore, our findings indicate that DHA induces mitochondrial dysfunction in A2780 cells.

Although it is well known that DHA treatment can alter cancer cell death and oxidative status, and mitochondrial function, it is unclear how these parameters are interconnected in ovarian cancer cells treated with DHA. In the present study, we dissected the intricate relationship between cell death induction, oxidative status, and mitochondrial function. We determined that DHA triggers ovarian cancer cell pyroptosis and leads to a decrease in mitochondrial membrane potential in a ROS-dependent manner, as stimulation with NAC abolished pore formation and mitochondrial membrane potential loss in cells stimulated with DHA. Herein, we also discovered that the effects of DHA on A2780 cell pyroptosis and mitochondrial function are dependent on caspase-1 activation, as A2780 pre-treatment with YVAD abrogates the effects of DHA on LDH release and mitochondrial dysfunction. Thus, our data suggest that the effects triggered by DHA on the ovarian cancer cell A2780 are mediated by increased ROS production and caspase-1 activation. Our data pave the way for discovering the mechanisms by which DHA leads to disrupted oxidative status, mitochondrial dysfunction, and pyroptotic cell death in ovarian cancer cells.

Almost all studies describing the effects of DHA on ovarian cancer involve in vitro models. To the best of our knowledge, Calbay and colleagues, Wang and collaborators, and West and others were the only researchers to explore ovarian tumor in vivo models to investigate DHA antitumoral properties [35, 41, 49]. This lack of in vivo analyses is also a limitation in the present study. We were efficient in investigating the effects of DHA on ovarian cancer cells in vitro. However, we were not able to validate our findings using additional experimental approaches, including 3D cell culture and mouse models, which would be key for investigating novel therapeutic targets for ovarian cancer and would enable researchers to explore DHA during potential clinical translations.

Therefore, herein we showed that DHA triggers ROS generation, mitochondrial dysfunction, and pyroptosis in ovarian cancer cells. We also described that these phenomena are dependent on the ovarian cancer cell oxidative status and caspase-1 activation. Our data aid in uncovering the cellular alterations triggered by DHA in ovarian cancer, a highly lethal malignancy worldwide.

## Conclusion

In this study, we demonstrated that the omega-3 fatty acid DHA induces pyroptotic cell death in A2780 human ovarian cancer cells. This process is characterized by early and late membrane pore formation, increased release of LDH, and activation of caspase-1 and caspase-3. We further show that DHA triggers a significant increase in intracellular ROS and mitochondrial superoxide levels, which are essential for the loss of mitochondrial membrane potential and the execution of pyroptosis. Additionally, DHA reduced mitochondrial content and severely impaired SRC, a key indicator of mitochondrial adaptability and energy metabolism, without altering basal or ATP-linked respiration.

Importantly, both ROS scavenging NAC and caspase-1 inhibition with YVAD were able to reverse DHA-induced membrane disruption, mitochondrial dysfunction, and cell death, establishing a mechanistic link between oxidative stress, inflammasome activation, and bioenergetic failure. These findings reveal, for the first time, that DHA-induced pyroptosis in ovarian cancer cells involves a non-apoptotic activation of caspase-3 and depends critically on mitochondrial dysfunction orchestrated by ROS and caspase-1 activity.

Together, our data contribute with novel insights into the mechanisms by which DHA disrupts ovarian cancer cell homeostasis and underscore the potential of targeting pyroptotic pathways and mitochondrial vulnerabilities as part of therapeutic strategies. Given the selective cytotoxicity observed in cancer cells and the growing interest in exploiting immunogenic cell death modalities, further studies in 3D culture systems and in vivo models are warranted to evaluate the translational relevance of DHA as an adjuvant or standalone anti-cancer agent in ovarian malignancies.

## Material and methods

### Cell culture and treatments

We cultured the human ovarian carcinoma cells A2780 at 37 °C and 5% CO_2_ in RPMI medium supplemented with 10% (v/v) fetal bovine serum (FBS) and 100 ng/mL Penicillin-Streptomycin (P-S) antibiotic mixture. We obtained A2780 cells from the Rio de Janeiro Cell Bank in June of 2018. Before we started conducting experiments, we treated A2780 cells with BM cyclin (Sigma–Aldrich) and verified *Mycoplasma* contamination using polymerase chain reaction (PCR). We also used PCR for A2780 cell line authentication.

Before treating cells, we transferred DHA (Sigma–Aldrich) to glass flasks, dissolved it in ethanol at 40 mM, and stored it at −20 °C. For A2780 cell treatment, we transferred the corresponding volumes of DHA solution to cylindrical glass tubes, left its ethanol to completely evaporate, and diluted the DHA in RPMI medium supplemented with 5% (v/v) FBS and 100 ng/mL Penicillin-Streptomycin antibiotic mixture. Then, we sonicated the medium/DHA solution for 10 min at room temperature and transferred it to the wells. For characterizing the phenomena induced by DHA in A2780 cells, we used 12.5, 25, 50, 100, and 200 μM.

In order to assess the importance of ROS levels in the context of A2780 DHA treatment, we used the ROS scavenger N-acetylcysteine (NAC)(Sigma-Aldrich). Prior to stimulating A2780 cells, we dissolved NAC in Milli-Q water at 600 mM and stored it at −20 °C. We treated A2780 cells with 5 mM of NAC 1 h before DHA treatment stimulation, maintaining NAC concentration until data acquisition.

To evaluate the effects of caspase-1 inhibition in the context of A2780 DHA treatment, we used the caspase-1-specific inhibitor Ac-YVAD-cmk (Sigma-Aldrich). Before stimulating A2780 cells, we dissolved YVAD in DMSO at 20 mM and stored it at −20 °C. We stimulated A2780 cells with 20 µM of YVAD 1 h before DHA treatment, and which concentration was maintained until data acquisition. The corresponding volume of DMSO was used as a vehicle control and was analysed along with other experimental conditions. In the experiments that included YVAD pre-treatment, we only used DHA at 50 μM.

### Cell viability assay

As a means of evaluating the effects of DHA on cell viability, we evaluated A2780 cell viability using 3-(4,5-dimethylthiazol-2-yl)-2,5-diphenyltetrazolium bromide— MTT (Life Technologies). We seeded A2780 cells in 96-well plates (5 × 10^3^ per well). After complete attachment, we stimulated A2780 cells with DHA at 12.5, 25, 50, 100, and 200 μM for 24 and 48 h. After stimulation, we exposed cells to 66.66 µg/mL of MTT, diluted in RPMI medium supplemented with 5% (v/v) FBS and 100 ng/mL P-S antibiotic mixture, for 1 h. Then, we dissolved insoluble formazan, a product derived from MTT mitochondrial metabolism, using 100 μL of DMSO. We obtained absorbance values using Spectramax M3 at 570 nm. We conducted cell viability data analysis using GraphPad Prism 8.0 software, considering absorbance presented by unstimulated (UNS) cells as 100% of viability and analyzing the other groups proportionally. As a non-transformed cell, we isolated PBMC-derived monocytes and treated these cells with the same DHA concentrations for 24 and 48 h. We obtained experimental control by exposing cells to 50% (v/v) of the cytotoxic agent DMSO (diluted in RPMI 5% FBS) for 24 h. The data were represented as a percentage of cell mitochondrial viability compared to UNS. We collected measurements in quintuplicate (*n* = 5) for each independent experimental replicate. Figures represent three independent experimental replicates. We used the mean as center value and standard deviation (SD) as error bars.

### Cell proliferation evaluation

To analyze the impacts of DHA on A2780 cell proliferation, we used the fluorescent probe carboxyfluorescein succinimidyl ester (CFSE) (Invitrogen), which correlates negatively with cell proliferation. We plated A2780 cells in 24-well plates (5 × 10^4^, or 2.5 × 10^4^, or 1.25 × 10^4^ per well, depending on the DHA stimulus time point). After 12 h for complete attachment, we incubated the cells with 5 μM of CFSE diluted in PBS 1× for 15 min at room temperature. Then, we exposed cells to FBS at 4 °C, as a means of chelating the extracellular CFSE molecules, and washed with PBS 1X twice. In the following step, we treated A2780 cells with DHA at 12.5, 25, 50, and 100 μM for 24, 48, and 72 h, collected the cells, washed once with PBS 1×, and fixed with paraformaldehyde 1%. We acquired samples using a FACS VERSE flow cytometer (BD Biosciences), collecting a total of 10,000 events per sample in the FITC channel (530/30 BP filter). We obtained data using V10 (Tree Star Inc.) software (Supplementary Fig. 1A, B). We used unstained cells and cells treated with 1 µM of colchicine as fluorescence and experimental controls, respectively. We represented data as mean fluorescence intensity (MFI). We collected measurements in triplicate (*n* = 3) for each independent experimental replicate. Figures represent three independent experimental replicates. We used the mean as center value and standard deviation (SD) as error bars.

### Cell cycle progression analysis

As a means of verifying the effects of DHA on A2780 cell cycle progression, we used propidium iodide (Sigma-Aldrich). We seeded A2780 cells in 12-well plates (10^5^ per well). After 12 h for complete attachment, we stimulated A2780 cells with DHA at 12.5, 25, 50, and 100 μM for 24 h. After treatment, we washed cells twice with PBS 1X and exposed cells to ethanol 70% (v/v) for 2 h at 4 °C. Then, we centrifuged cells once with PBS 1X (1000 × *g* for 10 min) and exposed to propidium iodide solution (20 μg/mL propidium iodide, 50 μg/mL RNAse, 0.1% (v/v) sodium citrate, 0.1% (v/v) Triton X-100) at room temperature for 30 min. PI was used to analyze the DNA content of the investigated cells, which enabled us to categorize events in different cell cycle phases. After incubation, we resuspended the cells in 1 mL of PBS 1X. We acquired samples using a FACS VERSE flow cytometer (BD Biosciences), collecting a total of 100,000 events per sample in the PE channel (586/42 BP filter) using linear scale. We obtained data using ModFit LT™ software. We used unstained cells and unstimulated cells as fluorescence and experimental controls, respectively. We represented data as a percentage of events per cell cycle phase. We collected measurements in triplicate (*n* = 3) for each independent experimental replicate. Figures represent three independent experimental replicates. We used the mean as center value and standard deviation (SD) as error bars.

### Cell death profile investigation

To investigate the impacts of DHA on A2780 cell death profile, we used the fluorescent probes Annexin V–FITC (Invitrogen) and propidium iodide. We plated A2780 cells in 24-well plates (5 × 10^4^ per well). After 12 h for complete attachment, we stimulated A2780 cells with DHA at 12.5, 25, 50, and 100 μM for 24 h. After treatment, we detached cells with trypsin and washed twice with PBS 1X at 1800 rpm centrifugation. Then, we diluted Annexin V-FITC to 10 µg/mL and propidium iodide to 20 µg/mL, both in binding buffer 1X (Invitrogen) (10 mM HEPES/NaOH pH 7.4, 140 mM NaCl and 2,5 mM CaCl_2_), resuspended A2780 cells with 25 µL of each solution and incubated these cells for 15 min in the dark at room temperature. After this step, we immediately acquired collecting a total of 10,000 events per sample using FACS Aria III flow cytometer (BD Biosciences) in the FITC (530/30 BP filter) and PerCP-Cy5.5 (700/54 BP filter) channels, collecting a total of 10,000 events per sample. We obtained data using FlowJo V10 (Tree Star Inc) software (Supplementary Fig. 1C). We used unstimulated cells as experimental control and unstained cells, and cells heated at 100 °C for 10 min and stained with Annexin V-FITC and propidium iodide solutions, separately or together, as fluorescence controls. We represented the data as a percentage of positive cells. We collected measurements in triplicate (*n* = 3) for each independent experimental replicate. Figures represent three independent experimental replicates. We used the mean as center value and standard deviation (SD) as error bars.

### LDH release analysis

We evaluated the effects of DHA on LDH release by A2780 cells using the LDH release Invitrogen commercial kit. We seeded A2780 cells in 96-well plates (2.5 × 10^3^ per well). After 12 h for complete attachment, we stimulated A2780 cells with DHA at 12.5, 25, 50, and 100 μM for 24 h. After treatment, we collected 50 µL of fresh A2780 cell supernatant and conducted the quantification of LDH release, following the protocol specifications. We collected the data using the spectrophotometer SpectraMax M3 (Molecular Devices) at 490 nm. We represented data as a percentage compared to the positive control, consisting of A2780 cells treated with the kit lysis buffer for 45 min before supernatant collection.

To investigate the impact of caspase-1 inhibition on A2780 LDH release in cells treated with DHA, we first stimulated cells with 20 µM YVAD for 1 h. Subsequently, we stimulated cells with 50 µM DHA for 24 h, maintaining the YVAD concentration. We also analysed LDH release using fresh A2780 cell supernatant following the kit specifications. We treated cells with the corresponding volume of DMSO to use as a vehicle control (Supplementary Fig. 2). Samples were analyzed using SpectraMax M3, and data were represented as a percentage compared to the positive control. We collected measurements in quintuplicate (*n* = 5) for each independent experimental replicate. Figure represents three independent experimental replicates. We used the mean as center value and standard deviation (SD) as error bars.

### Membrane pore formation assay

As a means of detecting whether DHA treatment leads to membrane pore formation in A2780 cells, we used the non-permeable fluorescent probe propidium iodide (Sigma–Aldrich). We seeded A2780 cells in Black/Clear Bottom 96-well plates (5 × 10^3^ per well). After 12 h for attachment, we exposed A2780 cells to 3 μg/mL of propidium iodide solution, diluted in RPMI medium free of phenol red and containing 5% FBS and 1% P-S, and immediately stimulated cells with DHA at 12.5, 25, 50 and 100 μM or only with 50 μM in the presence or absence of 5 mM NAC 1-h pre-treatment. We collected fluorescence measurements for 6 h with 1-h intervals between each read using 538 and 617 nm as excitation and emission wavelengths, respectively. We analysed data using GraphPad Prism 8.0 software. We represented data as relative fluorescence units (RFU). We collected measurements in triplicate (*n* = 3) for each independent experimental replicate. We collected measurements in quintuplicate (*n* = 5) for each independent experimental replicate. We used the mean as center value and standard deviation (SD) as error bars. Figure represents three independent experimental replicates.

### Microscopic analysis of membrane pore formation

To visualize the effects of DHA treatment on membrane pore formation in A2780 cells, we used the non-permeable fluorescent probe propidium iodide. We seeded A2780 cells in a 48-well plate (1 × 10^4^ per well). After 12 h for complete attachment, we exposed cells to 6 μg/mL of propidium iodide solution diluted in supplemented RPMI containing 5% FBS and 1% P-S and treated cells with DHA at 50 and 100 μM. We acquired images using the equipment CELLCYTE X^TM^ (CYTENA) and the software Cellcyte studio immediately after the treatment and 4, 8, 12, 16, 20, and 24 h after the stimulus. We collected measurements in quadruplicate (*n* = 4) for each independent experimental replicate. We included a representative figure of three independent experimental replicates.

### Caspase-1 activation assay

We investigated whether DHA influences caspase-1 activation in A2780 cells, using the fluorescent permeable probe FAM-FLICA Caspase-1 reagent (ImmunoChemistry Technologies). We seeded A2780 cells in 24-well plates (5 × 10^4^ per well). After 12 h for complete attachment, we treated cells with DHA at 12.5, 25, 50, and 100 μM for 24 h. Following treatment, we detached cells with trypsin, washed with PBS, and incubated with FAM-FLICA Caspase-1 reagent in RPMI without FBS at 37 °C for 30 min in the dark. After incubation, we washed the cells twice with Apoptosis buffer and immediately acquired samples on a FACS ARIA III flow cytometer (BD Biosciences) in the FITC channel (530/30 BP filter). We recorded a total of 10,000 events within gated events per sample to exclude debris and doublets. We analyzed data using FlowJo v10 software (BD Biosciences) (Supplementary Fig. 3). We used unstained and unstimulated cells as fluorescence and experimental controls, respectively. We represented data as mean fluorescence intensity (MFI). We collected measurements in triplicate (*n* = 3) for each independent experimental replicate. We used the mean as center value and standard deviation (SD) as error bars. Figure represents three independent experimental replicates.

### Caspase 3/7 activation analysis

As a means of analyzing if DHA copes with A2780 caspase-3/7 activation during lytic cell death, we used the with CellEvent® Caspase-3/7 Reagent (Thermo Fisher Scientific) and SYTOX® AADvanced™ solution. We plated A2780 cells in 24-well plates (5 × 10^4^ per well). After 12 h for complete adherence, we treated cells with DHA at 50 μM for 24 h. Following stimulation, we detached cells with trypsin, washed once with PBS, and incubated with CellEvent® Caspase-3/7 Reagent at a final concentration of 2 μM in RPMI at 37 °C for 25 min in the dark. Subsequently, we added 0.2 μL of 1 mM SYTOX® AADvanced™ solution to each sample and incubated for 5 min under the same conditions. We immediately acquired samples using FACS ARIA III flow cytometer (BD Biosciences). We detected fluorescence in the FITC channel (530/30 BP filter) for the CellEvent® Caspase-3/7 reagent and in the PerCP-Cy5.5 channel (695/40 BP filter) for SYTOX® fluorescsation settings using single-stained controls. We recorded a total of 10,000 events within the gated events per sample to exclude debris and doublets. We analyzed data using FlowJo v10 software (BD Biosciences) (Supplementary Fig. 4A). We used unstained and unstimulated cells as fluorescence and experimental controls, respectively. We represented data as percentages of positive cells. We collected measurements in triplicate (*n* = 3) for each independent experimental replicate. We used the mean as center value and standard deviation (SD) as error bars. Figure represents three independent experimental replicates.

### Early reactive oxygen species analysis

We assessed the early production of ROS in A2780 cells treated with DHA using the fluorogenic probe DCFDA (2′,7′-dichlorodihydrofluorescein diacetate) (Sigma–Aldrich). We seeded 2.5 × 10^3^ cells per well of black 96-well clear-bottom plates. After 12 h for complete attachment, we exposed cells to 4 μM of DCFDA for 45 min. Subsequently, we washed the cells twice and stimulated them with DHA at 12.5 μM, 25 μM, and 50 μM diluted in phenol red-free RPMI containing HEPES buffer. We immediately started collecting fluorescence readings using a spectrophotometer (*Spectramax M3, Molecular Devices*) for 4 h, with 10-min intervals between readings. We conducted the assay under light-protected conditions at 37 °C, and we measured fluorescence using excitation and emission wavelengths of 485 nm and 535 nm, respectively. We analysed the resulting data using GraphPad Prism software. We used unstained and unstimulated cells and cells treated with 600 μM of hydrogen peroxide as experimental controls. We represented data as as RFU. We collected measurements in quintuplicate (*n* = 5) for each independent experimental replicate. We used the mean as center value and standard deviation (SD) as error bars. Figure represents three independent experimental replicates.

### Late reactive oxygen species generation analysis

We quantified the generation of ROS in A2780 cells treated with DHA for 24 h using the permeable fluorescent probe CellROX Green (Invitrogen). We seeded A2780 cells in 24-well plates (5 × 10^4^ per well). After 12 h for complete attachment, we treated cells with DHA at 12.5, 25, and 50 μM for 24 h. After treatment, we stained cells with 2.5 μM of CellROX Green for 30 min at 37 °C. After this step, we washed the cells 3 times with PBS, detached the cells with trypsin, washed twice with PBS, and resuspended in PBS for acquisition of 10,000 events in BD FACSAria III in the FITC channel (530/30 BP filter). We analysed data using FlowJo V10 software (Supplementary Fig. 4B). We used unstimulated cells as experimental control and unstained cells, and cells treated with 50 µM of Menadione for 1 h before staining as fluorescence controls. We represented data as MFI. We collected measurements in triplicate (*n* = 3) for each independent experimental replicate. We used the mean as center value and standard deviation (SD) as error bars. Figure represents three independent experimental replicates.

### Microscopic investigation of ROS production and mitochondrial content

We conducted the microscopic analysis of ROS production and mitochondrial number in A2780 cells treated with DHA for 24 h using the fluorogenic probes CellROX Green and MitoTracker Red CMXRos (Invitrogen). We plated 3 × 10^4^ cells per well of 24-well plates containing 13 mm coverslips. After 12 h for complete attachment, we treated or not cells with 20 µM of YVAD or DMSO for 1 h and then stimulated them with 12.5, 25, and 50 μM of DHA for 24 h. Then, we incubated cells with a solution of MitoTracker Red CMXRos diluted in complete medium at a concentration of 100 nM at 37 °C for 30 min, protected from light. After staining, we washed cells three times with culture medium and fixed with 3.7% formalin diluted in pre-warmed medium at 37 °C for 15 min. Following fixation, we washed the cells three times with PBS under gentle agitation for 5 min each. We also stained the cells with a 1:5000 DAPI/PBS solution for 5 min at room temperature. Then, we washed the cells three more times with PBS and mounted the coverslips onto slides using anti-fading mounting medium (Agilent Technologies). We acquired the images using a laser fluorescence confocal microscope (*Leica TCS SP5*). We collected measurements in quadruplicate (*n* = 4) for each independent experimental replicate. We included a representative figure of three independent experimental replicates.

### Late mitochondrial superoxide generation

To verify whether DHA treatment triggers mitochondrial superoxide production after 24 h of DHA treatment, we used the permeable fluorescent probe MitoSOX^TM^ Red (Invitrogen). We seeded A2780 cells in 24-well plates (5 × 10^4^ per well). After completing 12 h for complete attachment, we treated cells with DHA at 50 μM for 24 h. Then, we washed the cells with PBS, detached the cells with trypsin, and washed again with PBS for staining with 500 nM of MitoSOX for 30 min at 37 °C. Afterwards, cells were washed twice with PBS and resuspended in PBS for acquisition of 10,000 events in BD FACSAria III in the PE channel (585/42 BP filter). We analysed data using FlowJo V10 software (Supplementary Fig. 4C). We used unstained and unstimulated cells as fluorescence and experimental controls. We represented data as MFI. We collected measurements in triplicate (*n* = 3) for each independent experimental replicate. We used the mean as center value and standard deviation (SD) as error bars. Figure represents three independent experimental replicates.

### Quantification of early mitochondrial superoxide production

We investigated the early production of mitochondrial superoxide by A2780 cells stimulated with DHA using the fluorogenic probe MitoSOX^TM^ Red (Invitrogen). We plated 2.5 × 10^3^ cells per well of black 96-well clear-bottom plates. After 12 h for complete attachment, we washed cells twice with PBS and exposed cells to 5 μM of MitoSOX for 10 min at 37 °C. After incubation, we washed cells twice with PBS and treated cells with 25 and 50 μM of DHA diluted in phenol red-free RPMI containing HEPES buffer for 4 h. We immediately started collecting fluorescence readings using *Spectramax M3, Molecular Devices* for 4 h, with 10-min intervals between readings. We performed the experiments under light-protected conditions at 37 °C. We measured fluorescence using excitation and emission wavelengths of 510 nm and 595 nm, respectively. We used unstained and unstimulated cells as experimental controls. We represented data as RFU. We collected measurements in quintuplicate (*n* = 5) for each independent experimental replicate. We used the mean as center value and standard deviation (SD) as error bars. Figure represents three independent experimental replicates.

### Evaluation of mitochondrial membrane potential

As a means of investigating whether DHA influenced A2780 cell mitochondrial membrane potential, we used the permeable fluorescent probe 5,5′,6,6′-tetrachloro-1,1′,3,3′-tetraethylbenzimidazolocarbocyanine iodide—JC-1 (Abcam). We seeded A2780 cells in 24-well culture plates (5 × 10^4^ per well). After 12 h for complete attachment, we pre-treated cells with 5 mM of NAC for 1 h and stimulated cells with 50 μM of DHA for 24 h, maintaining NAC concentration. Then, we washed the cells with PBS, detached the cells with trypsin, and washed again with PBS for staining with 2 μM of JC-1 for 15 min at 37 °C. After cell staining, we washed the cells with PBS and resuspended them in PBS for immediate flow cytometry acquisition. We acquired samples using a FACS VERSE flow cytometer (BD Biosciences), collecting a total of 10,000 events per sample in the FITC (530/30 BP filter) and PE (586/42 BP filter) channels. We analysed data using FlowJo V10 software (Supplementary Fig. 5). Unstimulated cells were used as experimental controls. Unstained cells and cells treated with 600 μM of hydrogen peroxide and with 20 μM of Carbonyl cyanide m-chlorophenyl hydrazone (CCCP) were used as fluorescence control. We represented data as a percentage of positive cells. We collected measurements in quadruplicate (*n* = 4) for each independent experimental replicate. We included representative data of three independent experimental replicates.

### High-resolution respirometry

To dissect the impact of DHA on mitochondrial function, we conducted high-resolution respirometry analysis. We seeded A2780 cells in six-well culture plates (5 × 10^5^ per well). After 12 h for complete attachment, we treated or not cells with 20 µM of YVAD or DMSO for 1 h and then stimulated them with DHA at 50 μM for 24 h. Then, we detached cells with trypsin and performed high-resolution respirometry using OROBOROS Oxygraph-O2K. We measured oxygen consumption rate (OCR) at 37 °C using 750 rpm continuous stirring in a 2 mL chamber. We used FBS-free RPMI for equipment calibration. After counting cells with a Neubauer chamber (diluted 10 times), we added the samples to the chambers in a concentration of approximately 1 × 10^6^ cells/mL, and we obtained the basal OCR after oxygen flux stabilization. We added the ATP-synthase inhibitor oligomycin at a concentration of 0.1 µg/mL, followed by titration of the uncoupler Carbonyl cyanide m-chlorophenylhydrazone (CCCP) at 14.4–21.6 µM and mitochondrial ETC complex I and III inhibitors, rotenone and antimycin A, at 0.5 µM and 1 µM, respectively. We calculated ATP-linked oxygen consumption by the difference between basal and proton-leaking OCR, and respiratory spare capacity by the difference between maximum capacity and basal OCR. We represented data as O_2_ flow per million cells. We collected measurements in septuplicate (*n* = 7) for each independent experimental replicate. We used the mean as center value and standard deviation (SD) as error bars. Figure represents three independent experimental replicates.

To determine whether caspase-1 inhibition impacted DHA-stimulated A2780 cells, we pre-treated cells with 20 µM of YVAD for 1 h, after which we stimulated cells with 50 µM of DHA for 24 h, maintaining YVAD concentration. Then, we performed high-resolution respirometry using OROBOROS Oxygraph-O2K, following the specifications detailed above. We treated cells with the corresponding volume of DMSO to use as a vehicle control (Supplementary Fig. 2). We also represented data as O_2_ flow per million cells. We represented data as MFI. We collected measurements in triplicate (*n* = 3) for each independent experimental replicate. We used the mean as center value and standard deviation (SD) as error bars. Figure represents three independent experimental replicates.

### Statistical analyses

We calculated sample size for each analysis prior to conducting experiments. We detected outliers using the Grubbs’ method. We investigated whether data followed a normal distribution using the Shapiro–Wilk normality test. We analysed parametric data using Student’s *t* test for comparing two experimental groups and ANOVA test and Tukey’s post-test for comparing three or more experimental groups, and presented data as column plots. We analysed non-parametric data using the Mann–Whitney test for comparing two experimental groups and the Kruskal–Wallis test and Dunn’s post-test for comparing three or more experimental groups, and presented data as box plots.

We assessed homogeneity of variance for each experiment. We represented differences compared to unstimulated (UNS) cells with asterisk(s) (*) and discrepancies between other groups with the number sign (#). These symbols were used following this rule: *p* < 0.05 (one symbol), *p* < 0.01 (two symbols), *p* < 0.001 (three symbols), and *p* < 0.0001 (four symbols). All the statistical analyses were performed using the GraphPad Prism 8.0 software. Figures represent independent replicates.

## Supplementary information

SUPPLEMENTARY VIDEO S1

SUPPLEMENTARY VIDEO S2

SUPPLEMENTARY VIDEO S3

SUPPLEMENTARY FIGURE 1

SUPPLEMENTARY FIGURE 2

SUPPLEMENTARY FIGURE 3

SUPPLEMENTARY FIGURE 4

SUPPLEMENTARY FIGURE 5

## Data Availability

All data included in this study are available upon request by contacting the corresponding author.
