# Supplementary figures and images for: The omega-3 DHA induces pyroptosis and mitochondrial dysfunction in ovarian cancer cells via ROS and caspase-1 activation
Source: Cell Death Discov. 2026 Jan 14;12:21. doi: 10.1038/s41420-025-02854-6 (PMC12804724; doi:10.1038/s41420-025-02854-6)

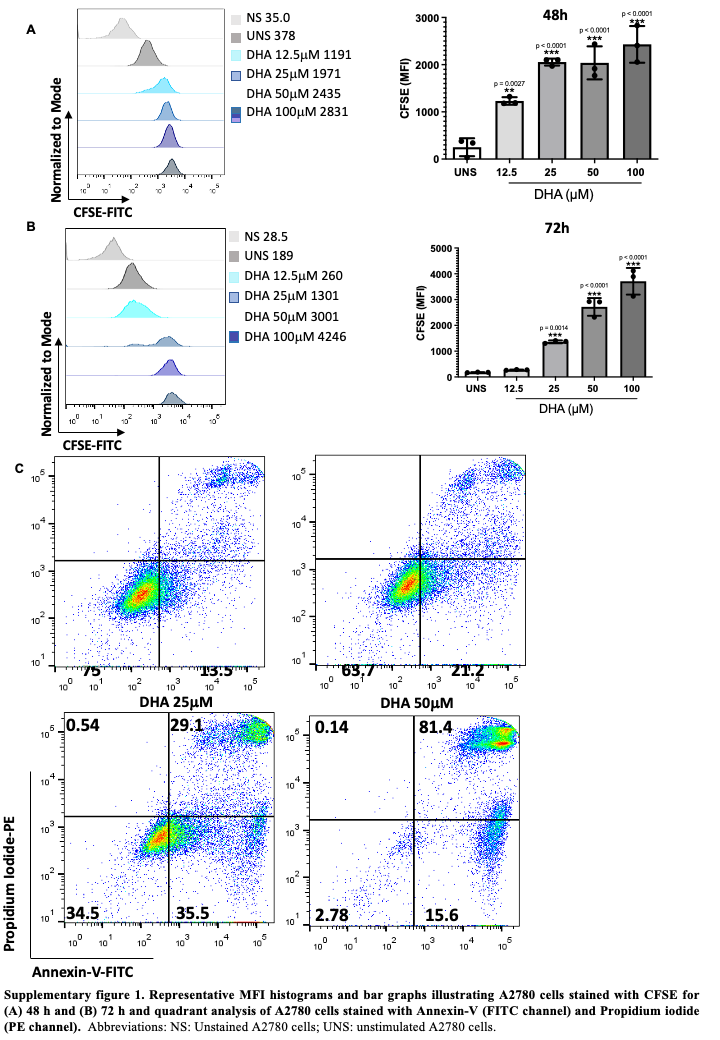

Supplement: Supplementary file 4 — SUPPLEMENTARY FIGURE 1 [file 41420_2025_2854_MOESM4_ESM.tif]

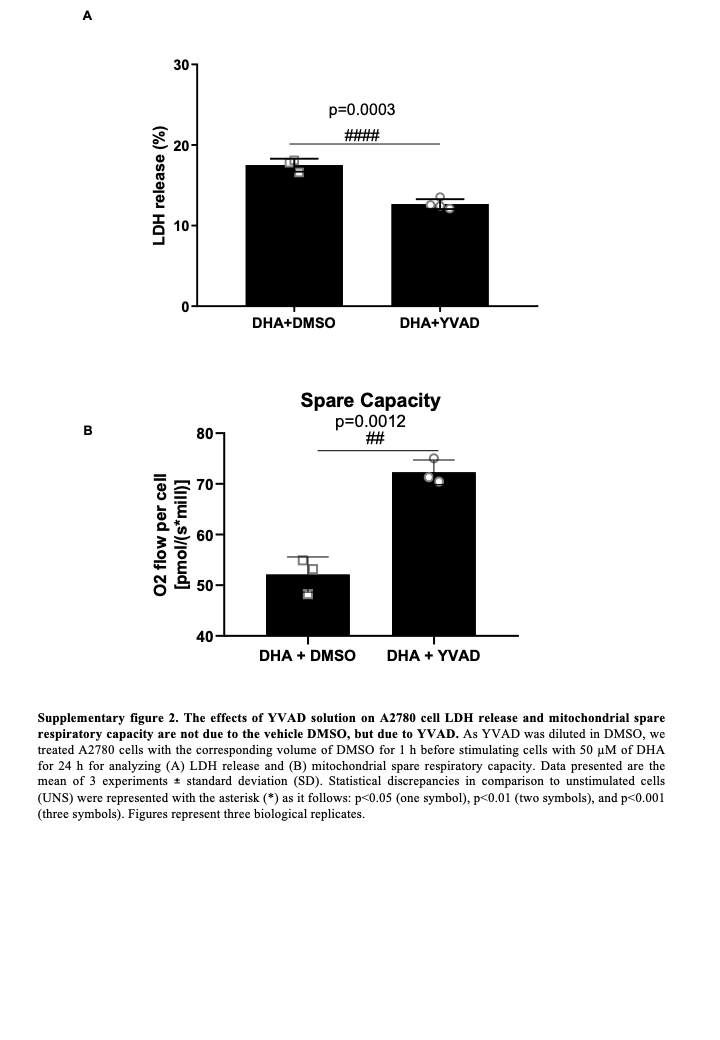

Supplement: Supplementary file 5 — SUPPLEMENTARY FIGURE 2 [file 41420_2025_2854_MOESM5_ESM.tif]

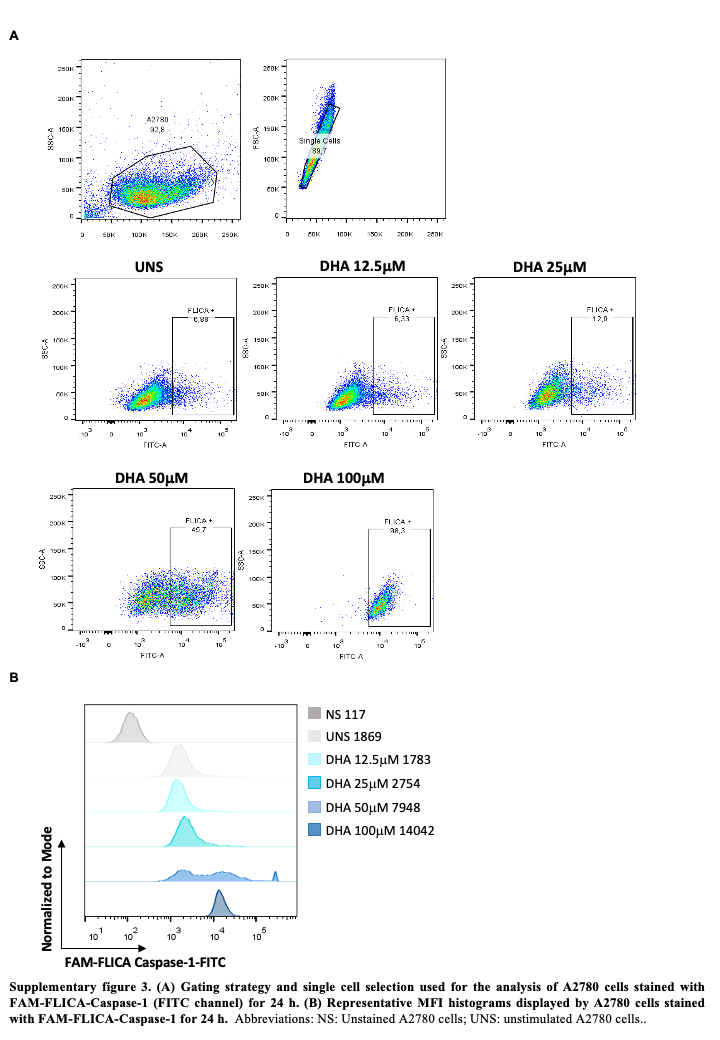

Supplement: Supplementary file 6 — SUPPLEMENTARY FIGURE 3 [file 41420_2025_2854_MOESM6_ESM.tif]

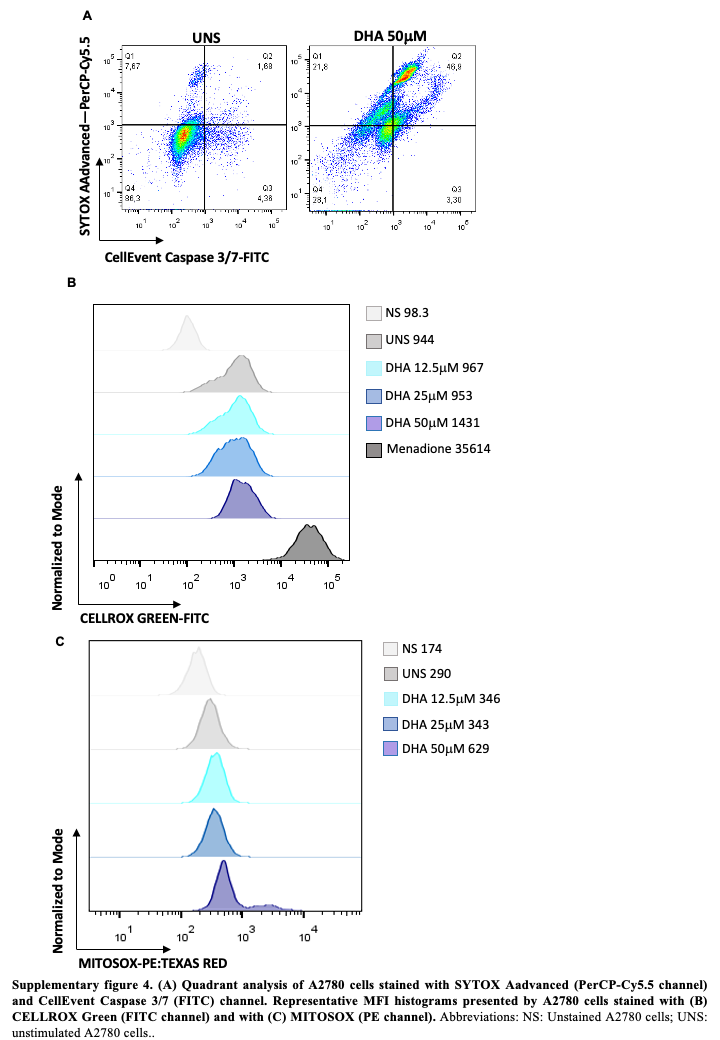

Supplement: Supplementary file 7 — SUPPLEMENTARY FIGURE 4 [file 41420_2025_2854_MOESM7_ESM.tif]

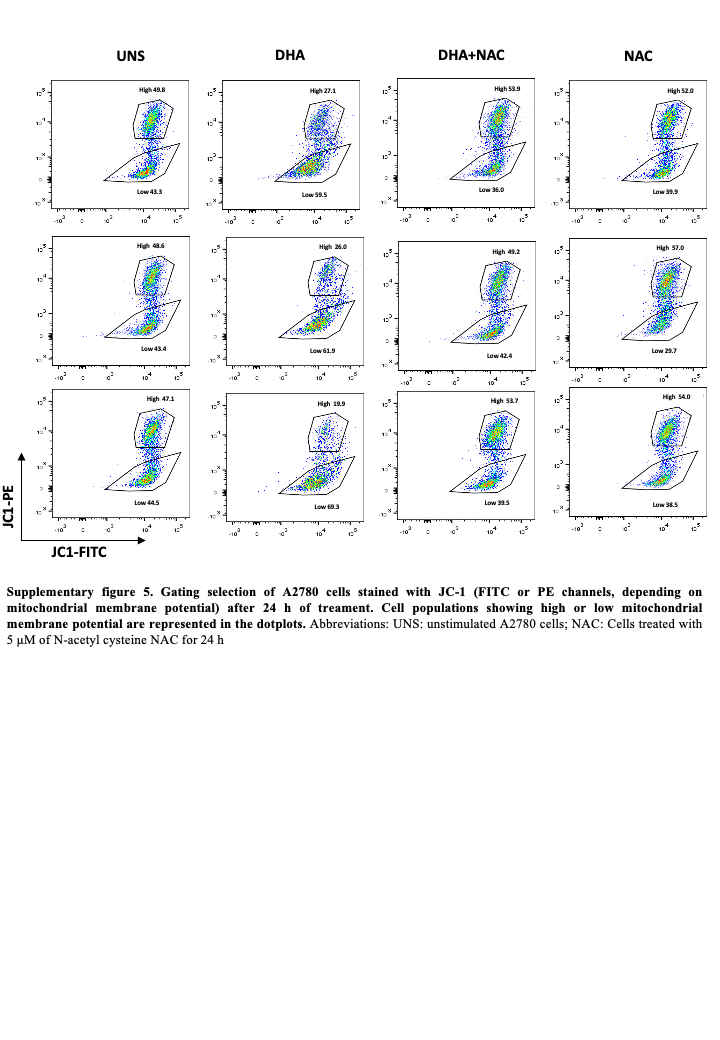

Supplement: Supplementary file 8 — SUPPLEMENTARY FIGURE 5 [file 41420_2025_2854_MOESM8_ESM.tif]
